# Health coaching with physical monitoring using health wearable (HCHW) to prevent non-communicable diseases (NCDs) in the middle-aged: a 4-arm randomized controlled trial protocol

**DOI:** 10.1186/s13063-025-09081-5

**Published:** 2025-10-08

**Authors:** Cyrus Tak Ka Tang, Cassie Cheuk Ling Lee, Eric Kam Pui Lee, Paul Kwok Ming Poon, Carmen Ka Man Wong, Xue Yang, Samuel Yeung Shan Wong, Benjamin Hon Kei Yip

**Affiliations:** The Jockey Club School of Public Health and Primary Care, The Chinese University of Hong Kong, School of Public Health Building, Prince of Wales Hospital, Shatin, New Territories Hong Kong

**Keywords:** Lifestyle intervention, Health coaching, Health watch, Cardiovascular disease (CVD), Non-communicable diseases (NCDs)

## Abstract

**Background:**

Non-communicable diseases (NCDs), also known as chronic diseases, contribute to a significant portion of the global disease burden and mortality. Unhealthy lifestyle factors play a major role in the development of NCDs, making middle age (35–59 years) a critical period for intervention, which can have a profound impact on delaying or preventing the incidence of chronic diseases. However, adopting lifestyle changes can be challenging, requiring interventions that consider psychological aspects and are easily incorporated into daily life.

**Methods:**

This 4-arm randomized controlled trial (RCT) aims to recruit 1000 middle-aged individuals without chronic diseases into four arms: health coaching (HC), health watch (HW), a combination of both (HC + HW), or waitlist control. The primary objectives are to assess the effectiveness of HC + HW compared to the waitlist group, as well as the effectiveness of HC-only and HW-only interventions. Secondary objectives include evaluating the impact on secondary outcomes and comparing the effectiveness between intervention groups. Life's Simple 7 (LS7) composite score will be assessed, along with biometric parameters and a risk assessment survey. Participants will undergo evaluations at 0 month, 3 months, and 6 months after randomization.

**Discussion:**

This 4-arm RCT will inform the effectiveness of HC and HW to support the community health by encouraging compliance to healthy lifestyle that prevent NCDs.

**Trial registration:**

This study was registered on Chinese Clinical Trial Registry on 26th February 2024 (no.: ChiCTR2400081193). All registered items are included within this manuscript.

**Supplementary Information:**

The online version contains supplementary material available at 10.1186/s13063-025-09081-5.

## Background

Non-communicable diseases (NCDs), commonly referred to as chronic diseases, constitute over 50% of the worldwide disease burden and cause 71% of global mortality annually [1–3]. Unhealthy lifestyles are the primary cause of NCDs, accounting for more than two-thirds of new cases [2]. Middle age, spanning 35 to 59 years, represents a critical juncture for intervention. For instance, the incidence of NCDs, including hypertension (HT) and diabetes mellitus (DM) nearly doubled among those aged 45–54 (from 15.7% to 34% for HT and 3.1% to 5.3% for DM) compared with the preceding age group of 35–44 in Hong Kong [4]. Research suggested that maintaining healthy habits at this “golden period” can have a profound impact on delaying or preventing the onset of NCDs typically manifesting after age of 60 [5, 6]. 

However, making lifestyle changes can be challenging and requires an understanding of individualized psychological factors, such as intention, motivation, expectations, reasons for change, and barriers to adopting or maintaining new habits [7]. Therefore, lifestyle interventions should be personalized to be effective. Promoting self-efficacy, self-determination, and self-responsibility is crucial for facilitating lasting behavior change [8]. Among such interventions, health coaching and health wearables hold significant promise.

Health coaching (HC) is a personalized intervention that facilitates individuals to achieve their health-related goals [9]. Health coaches assist individuals in problem solving and decision-making that foster positive changes, utilizing communication techniques including providing support, feedback and motivation, and listening [10] based on psychological theories and techniques of behavior changes, including the transtheoretical model [11], motivational interviewing (MI) [12], and cognitive behavioral therapy (CBT) [13–15].

Health coaching is widely used for patients with NCDs, significantly improving their chronic conditions, despite limited evidence from randomized controlled trials (RCTs). Nevertheless, HC has been shown to enhance adherence to healthy lifestyle choices and medication [16–19], increase physical activity [13], and improve motivation and personal satisfaction in weight loss [20]. Additionally, HC has significantly reduced systolic blood pressure (SBP) levels, improved diabetic bioindicators such as glycemic control (i.e., glycated hemoglobin (HbA1c) and fasting blood glucose (FBG)), and lowered low-density lipoprotein (LDL) level in patients with HT [21, 22], type II DM [23, 24], and hyperlipidemia [22], respectively. Notably, a recently published large-scale quasi-experimental study (*n* = 2,106,376) showed that HC can significantly improve HbA1c levels, lipid profiles, and body weight in pre-DM individuals [25].

Health wearables or health watches (HW) are some of the most popular health gadgets that typically incorporate health -tracking functions, including fitness monitoring, heart rate (HR) monitoring, oxygen saturation (SpO2) measurement, and sleep analysis. Due to their convenience, HW enable individuals to self-monitor their personal health [26]. In theory, an HW can serve as a personal monitoring tool, helping individuals understand their biological data and gain insights into their health [26]. Despite limited research, some studies have demonstrated the effectiveness of HW in helping individuals to adopt healthy behaviors. An RCT involving type 2 DM patients (*n* = 72) showed that providing activity trackers significantly increased participants’ weekly frequency of engaging in moderate to vigorous physical activity for at least 30 min on 1.5 days and increased participants’ daily step count by an average of 1255 steps per day [27]. Another RCT highlighted the broader impacts of HW on lifestyle behaviors and psychological well-being, such as self-actualization [28]. Systematic reviews have confirmed that wearable devices can significantly increase physical activity levels in individuals with chronic cardiometabolic diseases [29, 30].

While existing RCTs targeted patients with NCDs, there is a lack of HC or HW studies targeting populations at risk of developing NCDs. Additionally, despite the growing popularity of HW, there remains a lack of convincing evidence regarding their effectiveness. This study addresses these research gaps by investigating the effectiveness of HC, HW, and their combination in encouraging lifestyle changes. By combining HC with HW, the device should offer positive feedback to participants, encouraging them to adhere to the lifestyle modification advice given during health coaching [31]. This approach aims to improve both the effectiveness of and compliance with lifestyle changes. Thus, this study will provide valuable insights into designing interventions to foster healthy lifestyle, ultimately leading to NCD prevention.

## Objective and hypothesis

Participants will be randomized to one of the following arms: HC, HW, both interventions combined (HC + HW), or waitlist control. The primary objectives of this RCT is to investigate the effectiveness of each of the three intervention arms (HC + HW, HC, HW) in improving Life's Simple 7 (LS7) composite score (an integrated lifestyle score that assesses seven health metrics for cardiovascular health as defined by the American Heart Association (AHA)) [32], compared with waitlist control. Comparisons of effectiveness between the interventional arms (HC + HW, HC, HW) will be our secondary outcomes. We hypothesize that HC and HW, individually or in combination, will improve participants’ lifestyle behaviors, resulting in a higher LS7 score, when compared with waitlist control. We further hypothesize that combining HC and HW will result in superior effectiveness than either component alone.

## Trial design

This parallel, 4-arm RCT is designed as a superiority trial to assess the effectiveness of HC and HW, individually or in combination, in promoting a healthy lifestyle among middle-aged individuals without NCDs over a 6-month period. The participants will be aged 35–59 years, at risk of developing NCDs, but without any doctor-diagnosed NCDs. A total of 1000 eligible participants will be randomized equally into one of the four arms in a 1:1:1:1 ratio (*n* = 250 per group). The four arms are HC + HW, HC-only, HW-only, and control. While participants in the three intervention groups will receive their assigned interventions, the control group will not receive any intervention but will be followed up until the end of the trial. The primary assessment time point will be at 6 months after the intervention has started. The secondary assessment will occur at 3 months, during which participants will complete a shorter version of the survey.

## Method

### Study setting, recruitment, and consent

Recruitment is conducted between March 2024 and September 2024 across six study sites in Hong Kong, including four non-governmental organizations (NGOs). Participants are recruited through NGO mailing lists, social media, advertisements, and community talks. This trial is considered an extension of our ongoing cohort study which all participants receive HC + HW intervention. Therefore, the potential participant pool is considered sufficient. Informed consent is obtained from all participants prior to their inclusion in the trial. Registration is completed on the project website, where participants provide online consent. The research team is available by telephone or email to explain the consent form. A copy of the consent form is available online. After consent is obtained, the project health coach at the participant’s chosen site arranges a meeting with the participant. During this initial meeting, the health coach thoroughly explain the consent form and study procedures to ensure that participants fully understand the study and their rights. Participants are informed that they can withdraw from the study at any time.

**Fig. 1:**
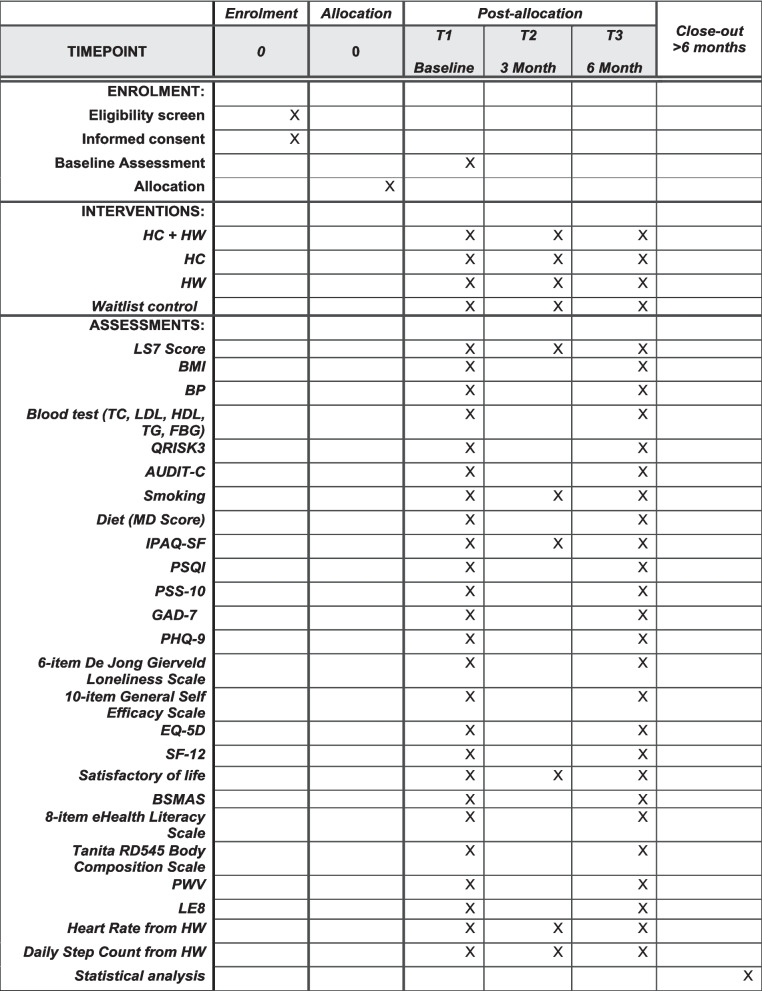
SPIRIT figure

Afterward, participants will complete a baseline assessment before randomization that evaluates their lifestyle across seven domains: physical activity, diet, sleep, stress, personal relationships, alcohol consumption, and smoking. Table 1 outlines the categorization of lifestyle domain levels. Identifying problematic lifestyle areas will guide coaching sessions and help with planning and setting health goals. All participants, regardless of their allocation, will receive a risk assessment session that reviews their lifestyle, anthropometric measurement, and blood test results in baseline. The Standard Protocol Items: Recommendations for Interventional Trials (SPIRIT) checklist was completed (see Additional file 1) and the SPIRIT figure (Fig. 1) is attached to ensure compliance with the SPIRIT reporting guidelines [33].

**Table 1:** Baseline risk assessment criteria of seven lifestyle domains

**Domain**	**Reference**	**Domain level**		
		**Poor**	**Intermediate**	**Ideal**
Physical activity	LS7 [91]	None—sedentary	1–149 min/week moderate or intense level of physical activity	≥ 150 min/week moderate or intense level of physical activity
Diet	MD [51]	MD score ≤ 7	8 ≤ MD score ≤ 9	MD score ≥ 10
Sleep	PSQI [53, 92]	14 < Global PSQI score ≤ 21	5 < Global PSQI score ≤ 14	0 ≤ Global PSQI score ≤ 5
Anxiety	GAD-7 [61, 63]	15 ≤ GAD-7 score ≤ 21	10 ≤ GAD-7 score ≤ 14	0 ≤ GAD-7 score ≤ 9
Personal relationship	6-item De Jong Gierveld Loneliness Scale [67]	5 ≤ Loneliness score ≤ 6	2 ≤ Loneliness score ≤ 4	0 ≤ Loneliness score ≤ 1
Alcohol consumption	AUDIT-C [49, 50]	AUDIT-C score ≥ 8	5 ≤ AUDIT-C score < 8	AUDIT-C score < 5
Smoking	LS7 [91]	Current smoker	Former smoker and quit within 12 months	Never smoked, or quit more than 12 months

### Inclusion and exclusion criteria

The inclusion criteria are as follows: (1) aged between 35 and 59 and (2) at risk of NCD development defined by at least two components of the following: 1. Body mass index (BMI) ≥ 23 kg/m^2^, 2. Diet (less than 4 components of healthy diet defined by LS7, please refer to “Primary outcome” for the detail), 3. Physical activity (< 150 min of moderate or intense level of exercise per week), 4. Smoking status (current smokers or former smokers quit within 12 months), 5. Family history of HT/DM/CVD in 1 st degree relative < 60 years of age, 6. Alcohol Use Disorders Identification Test-C (AUDIT-C) score ≥ 5, and 7. Total cholesterol (TC) level (6.2 mmol/L ≤ TC < 8 mmol/L).

The exclusion criteria are as follows: (1) unable to provide consent; (2) pregnancy; (3) do not understand Cantonese; (4) measured with blood lipid level (TC ≥ 8 mmol/L; LDL ≥ 5 mmol/L; triglyceride (TG) ≥ 5 mmol/L); (5) blood pressure (BP) (SBP ≥ 135 mmHg or diastolic BP (DBP) ≥ 85 mmHg); (6) QRISK-3 ≥ 15%; (7) with doctor-diagnosed chronic diseases and require medication (including DM, HT, CVD), chronic obstructive pulmonary diseases (COPD), depression, autoimmune diseases, and cancer.

### Intervention

The three intervention arms of the RCT consist of HC, HW, and their combination. All participants will receive their risk assessment profile at the beginning of the interventions. In the first arm, participants will receive both HC and HW as interventions. In the second arm, participants will receive only HC, and in the third arm, only HW. Participants in waitlist control will not receive any interventions during the 6-month study period following randomization.

After completion of all follow-up components, participants in the HC-only and waitlist control arm will be rewarded with the HW, while those in the HC + HW arm and the HW-only arm can keep their HW. Additionally, participants in the HW-only and control arm will receive HC after the 6-month assessment. This approach is intended to serve as an incentive to promote compliance and reduce dropout rates.

#### Health coaching (HC)

All health coaches have at least a bachelor’s degree in health, social science, psychology, or science. They were trained by an expert team, that includes a medical doctor, a certified health coach and psychologist, and a certified health and wellness coach trainer from the American Fitness Professionals and Associates (AFPA). The training program includes an 88-h HC training course and individual and group supervisions. Coaches acquire knowledge about HC fundamentals, health behavior, and behavior change techniques based on CBT, MI, and the transtheoretical model. Additionally, ongoing supervision and troubleshooting support are provided by the expert team during program implementation.

HC comprises the following psychometric components: goal-oriented, client-centered, partnership, health-focused, process, enlightening, and empowering [33]. The delivery mode, intervention components, duration, and frequency of follow-up sessions may vary [16, 34]. In general, each coaching session contains the following elements:

1. Discovery (for first sessions) or revision (for follow-up sessions): During initial sessions, coaches explore details regarding participants' lifestyle behavior using the questionnaire results. In follow-up sessions, they review and refine these points.
2. Creating awareness: Coaches help participants recognize their health-related behaviors and patterns.
3. Priorities: Participants and coaches jointly identify key areas for improvement.
4. Planning and goal setting: Based on insights, a tailored action plan is developed collaboratively.

Participants in the HC arm will receive different coaching intensity depending on their NCD risk level (please refer to Table 2 for the definition of NCD risk level). Low-risk and high-risk participants will receive 6 to 10 sessions (co-decided by the participant and the coach), and ≥ 12 sessions during the 6-month intervention period, respectively. In the first coaching session, health coaches will review the participants’ risk profiles and use a 10-point Likert scale to assess their priority, importance, readiness, and confidence to initiate lifestyle changes (higher scores indicate greater priority, importance, readiness, or confidence). The health coach will then employ various skills, adapting to specific situations, to co-design a personalized action plan with specific goals with the participants. Participants will also receive health education through the CORE program to acquire relevant knowledge to achieve these goals. The CORE program covers three topics: physical activity, diet, and mental health. Participants in the HC arm will be assigned to one of these topics, depending on their lifestyle domain risk and their preferences. Follow-up sessions will be conducted face-to-face, via phone, or through text messaging. The participant’s compliance and progress toward the established goals will be continually reviewed, and the action plan will be adjusted as needed.

**Table 2:** NCD risk assessment criteria

Assessment metric	Low risk	High risk	Suspected to have NCDs
Health watch	Yes	Yes	Nil
Health coaching	6–10 sessions	At least 12 sessions	Nil
CORE program	Yes	Yes	Nil
**A component**			
BMI^a^	23–24.9 kg/m^2^	≥ 25 kg/m^2^	–
Diet^a,b^	≤ 3 components	≤ 1 component	–
Physical activity^a^	1–149 min/week moderate level of exercise	None—sedentary	–
Smoking^a^	Former smoker, but quit within 12 months	Current smoker	–
TC^a^	TC ≤ 6.2 mmol/L	6.2 mmol/L < TC < 8 mmol/L	–
Family history of HT/DM/CVD in 1 st degree relative < 60 years of age	Yes	Yes	–
Alcohol use	5 ≤ AUDIT-C score < 8	AUDIT-C score ≥ 8	–
**B component**			
BP^a,c^	SBP 120–129 mmHg or DBP < 80 mmHg	SBP 130–134 mmHg or DBP 80–84 mmHg	SBP ≥ 135 or DBP ≥ 85 mmHg
FBG^a^	< 5.6 mmol/L	5.6–6.9 mmol/L	≥ 7 mmol/L
Blood lipids	–	–	TC ≥ 8 mmol/l or TG ≥ 5 mmol/l or LDL ≥ 5 mmol/l
CVD risk	–	–	QRISK3-2018 ≥ 15%
Definition	Not meeting “high-risk” criteria	At least 2 A components and 1 B component above	At least 1 B component above

^a^AHA simple 7 criteria risk factors [91]

^b^The five diet components are as follows: (1) 4.5 cups per day of fruits/vegetables, (2) servings of fish per week (3.5-oz servings), (3) 1500 mg/day of sodium, (4) 450 kcal (36 oz) per week of sweets/sugar-sweetened beverages, and (5) 3 servings per day of whole grains (1.1 g of fiber in 10 g of carbohydrate; 1-oz equivalent servings) [91]

^c^AOBP measurement [47]: Take mean BP readings with 1 min interval between readings. Use the average of this 3 readings. Measurement conditions: 1) Quiet room with comfortable temperature; 2) No smoking, caffeine, food, or exercise for 30 min before measurement; 3) Remain seated and relaxed for 3-5 min; 4) No talking by patient or staff during or between measurements. Measurement posture: 1) Sitting with back supported by chair; 2) Legs uncrossed, feet flat on floor; 3) Bare arm testing on table; mid-arm at heart level

#### Health watch (HW)

Participants assigned to the HW group will receive a Huawei Watch Fit 2 smartwatch (Huawei Technologies Co., Ltd., China). The device tracks various health metrics, including physical activity, daily step counts, and heart rate (HR), serving as an objective tool to assess participants’ overall health status. Participants are encouraged to wear the HW daily. The Huawei Watch Fit 2 underwent internal validation by an optoelectronics expert. The accuracy of SpO2 and HR measurements from Huawei Watch Fit 2 were comparable to those from the COMEN-C502 (Shenzhen Comen Medical Instruments Co., Ltd., China) and the BCI-Autocorr PLUS Digital Pulse Oximeter (Smiths Medical Inc., USA) showing a mean absolute error difference of 2 BPM for HR and 2.2% for SpO2. The HUAWEI Health application, which accompanies the smartwatch, includes built-in workout programs and allows users to set physical activity targets. Participants are required to synchronize their HW with the project cloud. Reminders will be sent to participants who fail to synchronize or without HW data. Technical staff are available via text messaging to assist with any technical issues related to the HW.

#### Health coaching and Health watch (HC + HW)

Participants in the HC + HW group will receive 6 months of HC follow-up along with a Huawei Watch Fit 2 smartwatch. In each follow-up session, health coaches will also utilize data captured by the HW to enrich the HC context.

#### Waitlist control

Participants in the waitlist control group will not receive any of the abovementioned interventions during the 6-month follow-up period.

### Sample size calculation

We used a web-based multi-arm clinical trial sample size calculator [35] for the sample size (or power) calculation. In this four-arm trial, with three experimental treatments and a waitlist arm, the hypotheses to be tested will be:

$${H}_{k}:{\tau }_{k}={\mu }_{k}-{\mu }_{0}\le 0,\hspace{0.25em}k=1,\dots ,3.$$

The *global null hypothesis*, ${H}_{G}$, will be:

$${\tau }_{1}={\tau }_{2}={\tau }_{3}=0.$$

The *global alternative hypothesis*, ${H}_{A}$, will be:

$${\tau }_{1}={\tau }_{2}={\tau }_{3}={\delta }_{1}.$$

The *least favorable configuration* for experimental arm $k$, $LF{C}_{k}$, will be:

$${\tau }_{k}={\delta }_{1},\hspace{0.25em}{\tau }_{1}=\cdots ={\tau }_{k-1}={\tau }_{k+1}=\cdots ={\tau }_{K}={\delta }_{0}.$$

Here, *δ*_1_ represents the interesting treatment effect, which was defined as LS7-score difference of 0.44 with a common standard deviation (SD) of 1.57, and *δ*_0_ represents uninteresting treatment effects, which was defined as the smallest mean difference between the intervention and control arm that would make the intervention clinically significant, and was set to be 0 [35]. Due to a lack of evidence, we will not explicitly test the interaction effect of HC and HW. The combined intervention will be analyzed as a distinct arm compared to the control, rather than a factorial combination. Under the global alternative hypothesis, a sample size of (*N* = 720) will have a disjunctive power (power to reject at least one null hypothesis) of 0.901 after Bonferroni’s correction. This estimation used a balanced target group allocation and a 0.05 significance level. The assumed mean and SD were based on our ongoing cohort study of 1862 participants who received HC + HW interventions. The participants in this cohort study have the same eligibility criteria as those in the proposed trial. According to previous studies, an improved LS7 score of 0.44 is considered a significant health improvement [36–38]. We propose a target sample size of 1000, allowing for a dropout rate of up to 28%. However, the observed dropout rate in the cohort study is much lower (3.8%).

### Randomization and blinding

Eligible participants will be allocated to one of the four arms using permuted block and stratified randomization by sex (male/female), age group (35–50 and 50–59), and six study sites in a 1:1:1:1 ratio (with 250 participants in each arm). The randomization lists will be generated by an independent statistician, employing block sizes that vary between 8 and 16, which will remain undisclosed to the research team. The randomization lists will be kept in a password-encrypted file, accessible only to an impartial researcher from the research team. This researcher will handle the allocation procedure and communicate the results to the project health coaches via an online portal. The online system will display only de-identified eligible participants, identified by project ID, who have completed the baseline assessments. Each morning during the recruitment phase, this researcher will assign eligible participant to intervention arm according to the randomization lists. Project health coaches will be able to identify their allocated participants as indicated in the online portal. Subsequently, the health coach will schedule the participant’s first intervention session and inform them of their assigned intervention arm. Due to the nature of the intervention, neither participants nor project health coaches will be blinded to the allocation. However, the research team and the principal investigator (PI) will remain blinded to group assignments. Statisticians responsible for data analysis will remain blinded to group assignments until the analysis is complete. Participants who experience serious adverse events (SAEs) during the trial will be unblinded to the PI or research team.

### Data collection and management

All data will be collected via the project’s online administrative portal. All assessment survey conducted by participants are implemented with range checks for data entry validation. Health assessment data will be entered by project health coaches. Access to the portal backend is restricted to the PI and the research team. The raw personal data of participants will remain strictly confidential and will only be accessible to designated research staff and health coaches. All personal identifiers (e.g., name, HKID, telephone number, WhatsApp) will be removed from the analytical data and replaced with pseudo-identification numbers to ensure participant anonymity. Physical copies of sensitive documents, if any, will be stored in a double-locked filing cabinet in the research office, with access restricted to authorized personnel only. Electronic data will be stored securely on the servers managed by the Information Technology Services Centre (ITSC) of The Chinese University of Hong Kong (CUHK). These servers are protected by firewalls, password access controls, and are monitored 24 h a day. Data access will be restricted to the principal investigator and delegated research staff. All data transfers will utilize encrypted channels. All data will be retained in accordance with institutional guidelines and will not be shared with unauthorized individuals. Data security and confidentiality protocols will be regularly reviewed and updated as necessary. All blood samples will be used exclusively for biochemical testing and will not be retained after the tests have been completed. All blood samples collected from participants at baseline and at the 6-month assessment will be used solely for biochemical testing by private Hong Kong Laboratory Accreditation Scheme (HOKLAS)-accredited laboratories [39]. Samples will not be stored or retained after testing.

As the trial involves low-risk participants and low-risk interventions, a data and safety monitoring committee is not required. The PI will be responsible for monitoring the progress and implementation of the trial. Additionally, the PI will have regular biweekly meeting with the research team and audits throughout the study to ensure adherence to the trial protocol. No interim analysis will be conducted. The PI and research team will meet with NGO representatives monthly for the first 6 months, then quarterly, to review the progress of the trial.

### Severe adverse events (SAEs)

Although the trial is considered to have a relatively low risk of SAEs, any SAE that occurs will be overseen and reviewed by the project’s clinician. SAE are defined as any undesirable medical occurrence that is fatal, life-threatening, results in hospitalization or prolongation of existing hospitalization, causes persistent or significant disability/incapacity, or results in a congenital anomaly/birth defect [40]. We will collect these SAEs from health coaches and participant self-reports. The clinician will assess each case to determine whether participants should be withdrawn from the trial or temporarily suspended from the intervention. All SAE cases will subsequently be reported to the research ethics committee and will be reported in future publications regarding the trial results. Participants who experiences SAE will not receive any ancillary or post-trial care.

## Outcomes measurement

Blood tests, BP, anthropometric measurements, and a health assessment questionnaire, will be conducted both at baseline and 6 months after randomization. Additionally, participants will receive a shorter follow-up questionnaire at 3 months.

### Primary outcome

#### LS7 score

The primary outcome is the LS7 score at 6 months compared with baseline, analyzed as the mean ± SD and mean difference. The LS7 scores consists of seven health metrics including physical activity, diet, smoking, BMI, TC, BP, and FBG [32]. Each LS7 score metric is evaluated as poor (0 point), intermediate (1 point), or ideal (2 points). The scoring scheme is as follows: physical activity (ideal: ≥ 150 min/week moderate or intense level of exercise, intermediate: 1–149 min/week moderate to vigorous level of exercise, poor: none—sedentary); diet (ideal: 4–5 components, intermediate: 2–3 components, poor: 0–1 components; the diet components refer to the following: at least 4.5 cups per day of fruits/vegetables, or at least 3.5-oz servings of fish per week, or less than 1500 mg/day of sodium consumption per day, or less than 450 kcal (36 oz) per week of sweets/sugar-sweetened beverages, or less than 3 servings per day of whole grains); smoking (ideal: never smoked or quit more than 12 months ago, intermediate: former smoker, but quit within 12 months, poor: current smokers); BMI (ideal: ≤ 22.9 kg/m^2^, intermediate: 23–24.9 kg/m^2^, poor: ≥ 25 kg/m^2^); TC (ideal: < 6.2 mmol/L, intermediate: 6.2–7.9 mmol/L, poor: > 7.9 mmol/L); BP (ideal: SBP < 120 mmHg and DBP < 80 mmHg, intermediate: SBP: 120–134 mmHg or DBP: 80–84 mmHg, poor: SBP ≥ 135 mmHg or DBP ≥ 85 mmHg); and FBG (ideal: < 5.6 mmol/L, intermediate: 5.7–6.9 mmol/L, poor: ≥ 7.0 mmol/L). These individual scores are then added together to form a composite score that ranges from 0 to 14 [41–44]. The LS7 score was validated against the Pooled Cohort Equation (PCE), which was considered as the golden standard tool on predicting 10-year CVD risk, on prediction to CVD and all-cause mortality [45]. A higher LS7 score signifies better cardiovascular health [46]. LS7 score will be further categorized into poor (0–4), intermediate (5–9), and ideal [10–14, 41].

### Secondary outcomes

#### Biometric data

All biometric data, including SBP, DBP, and biochemical markers (FBG, TC, LDL, high-density lipoprotein (HDL), and TG), were collected at baseline and at the 6-month assessment time point. All parameters will be analyzed and presented as mean ± SD. Analyses will compare the differences between baseline and 6-month values. In addition, all biometric data will be further categorized based on its clinically significant thresholds [47–50].

SBP and DBP are measured by a validated automated office BP (AOBP) device (Microlife WatchBP Office 2G, Microlife Corp., Taiwan). The BP measurement procedures follow the standards of 2021 European Society of Hypertension practice guidelines for office BP measurement [47]. The BP device automatically takes three BP readings within a 1-min interval and provides a mean BP value for analysis, in the absence of healthcare workers to prevent white-coat effects. The device also does not display BP numbers during the 3-min measurement period.

In addition, the 10-year risk of developing CVD of participants at baseline and at the 6-month assessment timepoint will be estimated by QRISK3 score, using participants’ BP, blood cholesterols, BMI, and medical history. QRISK3 estimates an individual’s risk of developing CVD within the next 10 years [51].

#### Lifestyle behavior

Alcohol use will be measured with AUDIT-C at baseline and at the 6-month assessment. AUDIT-C is recommended by the World Health Organization (WHO) to understand the participants’ alcohol consumption risk (e.g., low, hazardous, harmful alcohol use, or alcohol dependence) [52]. Among the shorter versions of the Chinese-translated Alcohol Use Disorders Identification Test (Ch-AUDIT), the AUDIT-C demonstrates the best overall performance, with an area under the curve (AUC) 0.901 compared with 0.898 using Ch-AUDIT [50].

Diet will be assessed by a self-designed questionnaire at baseline and at 6-month assessment. The self-designed questionnaire took references from the Mediterranean diet (MD) and Dietary Approaches to Stop Hypertension (DASH) diet. Dietary frequency and portion intake of grains, fresh fruits, cooked vegetables, raw vegetables, poultry, red meat, processed meat, fish, milk/dairy, beans and nuts, sugar, and wine will be assessed [53]. In addition, a 14-item MD score will be calculated to standardize participant’s dietary habit. A higher score represents better adherence to MD and is associated with lower BMI. 

Physical activity level, including Metabolic Equivalent of Task (MET), total weekly physical activity level, weekly walking duration, and daily sedentary time, will be measured by the Chinese version of International Physical Activity Questionnaire-Short Form (IPAQ-SF) at baseline, at 3-month assessment, and at the 6-month assessment [54]. It contains 7 self-reported items that record how much time during the past 7 days the participant spends on (a) vigorous-intensity activity (e.g, aerobics), (b) moderate-intensity activity (e.g, leisure cycling), (c) walking, and (d) sitting, respectively. A systematic review reported that the IPAQ-SF demonstrated good psychometric properties [55] and has been used among Hong Kong Chinese [54]. All parameters related to physical activity levels will be categorized according to the WHO standards [56].

Sleep quality and disturbances will be measured with the Pittsburgh Sleep Quality Index (PSQI) at baseline and at the 6-month assessment. The PSQI is a 19-item self-rated questionnaire that generates seven components scores, summed to yield a global score ranging from 0 to 21. A higher score indicates higher sleep disturbance [57].

Smoking status of the participants was assessed by categorizing individuals as current smokers (1–10 cigarettes per day, 11–20 cigarettes per day, or more > 20 cigarettes per day), past smokers (having quit smoking within the past 12 months or more than 12 months ago), or non-smokers at baseline, at 3-month, and at the 6-month assessment. The distribution of smoking status at each assessment will be analyzed and compared with that at baseline.

#### Mental health parameters

Perceived stress will be measured with the 10-item Perceived Stress Scale (PSS-10) at baseline and at the 6-month assessment [58, 59]. Participants are asked to rate their feelings and thoughts during the last month on a 5-point Likert scale from 0= never to 4 = very often. It has been used in the Chinese population [60, 61]. The PSS-10 is a validated questionnaire to measure stress in the Chinese community [62, 63].

Anxiety will be assessed by the 7-item Generalized Anxiety Disorder Scale (GAD-7) at baseline and at the 6-month assessment [64]. It is a brief, self-reported scale for identifying probable cases of GAD. The Chinese version has been validated and used in the Chinese population [65, 66]. Participants are asked how often they were bothered by each symptom in the past 2 weeks on a 4-point Likert scale from 0= not at all to 3 = nearly every day. The GAD-7 total score ranges from 0 to 21, with higher scores indicating higher level of anxiety. A score of 0 to 4, 5 to 9, 10 to 14, and 15 to 21 represent minimal, mild, moderate, and severe anxiety, respectively.

Depression will be measured by the 9-item Patient Health Questionnaire-9 (PHQ-9) at baseline and at the 6-month assessment [67]. The Chinese version has been validated and used in the Chinese population [68, 69]. Participants are asked to rate how often they have been bothered by the symptoms in the past 2 weeks on a 4-point Likert scale from 0= not at all to 3 = almost every day. The total score ranges from 0 to 27, with higher score indicating greater level of depression. Scores of 0 to 4, 5 to 9, 10 to 14, 15 to 19, and 20 to 27 represent minimal, mild, moderate, moderately severe, and severe depression, respectively.

Loneliness of participants will be assessed by the validated Chinese version of the 6-item De Jong Gierveld Loneliness Scale. The results will be summed, and a lower total score means more loneliness [70].

Self-efficacy will be measured by the 10-item General Self Efficacy Scale [71]. It assesses self-beliefs to cope with a variety of difficult demands in life. Responses are rated on a 4-point Likert scale from 1= not true at all to 4 = exactly true. It has been used among Chinese [72]. The Chinese version of the 10-item General Self Efficacy Scale has been validated [73].

#### Quality of life

Quality of life will be measured using the EQ-5D, SF-12, and Satisfaction with Life Scale. The EQ-5D and SF-12 will be assessed at baseline and at the 6-month assessment, while the Satisfaction with Life Scale will be administered at baseline, 3 months, and 6 months. The EQ-5D is a questionnaire consisting of five dimensions and a Visual Analog Scale (VAS) that allows individuals to rate their perceived health status on a scale from 0 to 100 [74]. The SF-12 is a questionnaire comprising 12 questions designed to evaluate both the physical and mental health of individuals. It has been validated for use within the Chinese population [75]. Satisfactory of life scale of participants will be assessed by a single item ranging from 0 to 10, with higher scores indicating greater life satisfaction.

#### Factors for exploratory analysis

Social media addiction will be measured with the Bergen Social Media Addiction Scale (BSMAS) at baseline and at the 6-month assessment. The BSMAS is a 6-item self-reported scale with a total score ranging from 6 to 30. A higher score reflects higher social media addiction [76].

The 8-item eHealth Literacy Scale will be used to measure participant’s perceived skill to use information technology for health-related reasons at baseline and at the 6-month assessment [77].

Body composition will be measured with the Tanita RD545 body composition scale at baseline and at the 6-month assessment (Tanita Corp., Japan). The skeletal muscle mass measured by this machine was validated against Lunar iDXA (GE HealthCare Technologies Inc., USA) in older adults [78]. Information on body fat, muscle mass, and body water will be collected.

Pulse wave velocity (PWV) will be measured with the SMT Medical-Vicorder (80 beats medical, Germany) at baseline and at the 6-month assessment. The Vicorder machine is a non-invasive measurement of vascular health and has demonstrated sufficient accuracy and validity in measuring PWV when compared to the SphygmoCor machine (Cardiex Ltd, Australia), which is a validated non-invasive PWV measurement machine [79, 80]. Participants who took part in the project in the Sha Tin, N.T. study site, will have access to PWV measurement, and it will be conducted within the same district. A total of 167 participants will be invited for PWV measurements.

The Huawei Watch Fit 2 will be provided to participants in arm 1 and arm 3. The watch will collect parameters including HR and daily step count. Step count data will be averaged weekly and presented as mean ± SD. HR data will be used to identify periods of physical activity and to determine whether participants have reached specific HR zones associated with physical activity. As only participants in arm 1 and arm 3 will receive the HW, data will be compared within each arm and between arms 1 and 3 for the periods of 0–3 months and 3–6 months.

The Life's Essential 8 (LE8) composite score at baseline and at 6-month assessment will be calculated using lifestyle and biometric data such as diet, physical activity, sleep duration, smoking status, BMI, BP, FBG, and blood lipid levels. The LE8 score ranges from 0 to 100, with higher scores reflecting a healthier lifestyle and better cardiovascular health [81]. This score is based on the latest guidelines from the AHA for cardiovascular health. Our study was planned before LE8 became available. Therefore, it is included as an exploratory variable.

For continuous secondary outcome measurements—including biometric data (FBG, TC, LDL, HDL, TG, SBP, DBP, and QRISK3 score), lifestyle parameters (AUDIT-C score, physical activity parameters, MD score, PSQI score), mental parameters (PSS-10 score, GAD-7 score, PHQ-9 score, 6-item De Jong Gierveld Loneliness Scale, 10-item General Self Efficacy Scale), quality of life (SF-12, EQ-5D, satisfaction of life scale), and exploratory parameters (BSMAS, 8-item eHealth Literacy score, body composition data, Huawei Watch Fit 2-related parameters, LE8 score), the 6-month and, if available, 3-month assessment data will be compared with baseline values. Data will be presented as mean ± SD. For categorical secondary outcomes, the distribution of each outcome at every assessment time point will be analyzed and compared to baseline. Descriptive statistics (such as counts and percentages) will be presented for each category.

## Statistical analysis

To address the primary objective, the global comparison test, we will first conduct ANCOVA analysis, using baseline primary outcomes measures (LS7 score) as the covariates and the 6-month measurement as the outcome. For objective 2, we will conduct the same statistical analysis to compare arm 1 (HC + HW) vs arm 4 (waiting group). Similar analyses will be conducted for the other objectives. We will use Bonferroni correction to control the family-wise type I error rate at 0.05.

For exploratory analyses, we will conduct similar analytical plan for the secondary outcomes and subgroup analyses to investigate baseline influence. Post hoc tests will be used to investigate which pairs of levels within a factor differ after an overall (main effect) difference has been established.

We will use linear mixed models (LMMs) to address missing data. LMMs use likelihood-based estimation and are robust in addressing various types of missing data, including missing at random (MAR). Unlike imputation methods, LMMs leverage all information available in the current data, including fixed and random effects which minimizes potential biases related to imputation [82].

Statistical analysis will be performed in R, which is a free-of-charge computer software. Both intention-to-treat (ITT) and per-protocol approach will be used when comparing results across the 4 arms. For the ITT analysis, all randomized participants will be included. For the per-protocol analysis, only participants who have completed all assessments will be included.

## Discussion

This is the first 4-arm RCT that aims to evaluate the effectiveness of HC and HW in encouraging healthy lifestyles that prevent NCD development among at-risk middle-aged individuals. The study design, which includes a combination of HC and HW, HC-only, HW-only, and a waitlist control group, allows a comprehensive assessment of these interventions both individually and simultaneously.

Although both HC and HW have varying degrees of effectiveness for patients with NCDs (including CVD, DM, and HT) in lifestyle modification and disease management [10, 16, 21–24, 29, 30], the evidence supporting the effectiveness of HW is less convincing [83, 84]. Whether the inclusion of HW can enhance the effectiveness of HC remains unknown. Additionally, most prior studies have only recruited patients with NCDs, with fewer studies focusing on populations that are at-risk of NCD development. Recently, significant improvement in HbA1c, body weight, and lipid levels were observed in pre-DM patients after receiving the UK National Health Service Diabetes Prevention Programme (NHS DPP), which involves 9-month intensive lifestyle counseling and behavior-change interventions [25, 85]. However, the absence of a control group and the potential inclusion of participants on medication may have introduced confounding factors, limiting the accurate assessment of the intervention’s effectiveness. The NHS DPP was found to be cost-effective by a few studies [86–88], suggesting that implementing a similar large-scale prevention program could be both beneficial and economical. While the NHS DPP employs nurses, dietitians, and health coaches to deliver interventions, our program relies solely on health coaches, which should reduce intervention costs. Nevertheless, we must also consider the mode, frequency, and duration in delivering the intervention.

The anticipated findings from this RCT could have significant implications for public health strategies aimed at preventing NCDs. Similar to other developing regions, the Hong Kong government recognizes the significance of primary health care and has implemented the Co-Care pilot scheme to enhance awareness and screening for HT, DM, and hypercholesterolemia [89]. By demonstrating the effectiveness of HC and HW, either alone or in combination, this RCT will provide a standardized, scalable, and sustainable lifestyle intervention that can be integrated into routine primary care clinical practice, specifically targeting at-risk populations. The use of wearable technology for continuous health monitoring, coupled with personalized HC, may offer an expandable and sustainable approach to promoting healthy lifestyle changes. This could reduce the burden on healthcare systems by lowering the incidence of NCDs and decreasing the need for medical interventions and hospitalizations [90]. In the future, a cost-effectiveness analysis may be conducted to estimate the reduce in the healthcare burden arose from preventing NCDs, showing a long-term economic impact of implementing HC and HW. The future cost reduction could be induced from a lower rate of high blood glucose level, high BP, and 10-year CVD risk of the intervention arms compared to the control group with the application of simulation model [91–93]. The necessary variables could be directly obtained from the biometric parameters and QRISK3 score measured in this study. These findings could inform policy-making, leading to the development of guidelines and programs that leverage technology and personalized coaching to enhance public health outcomes. The success of such interventions could also stimulate further research into the long-term benefits and cost-effectiveness of integrating wearable devices and HC into preventive healthcare. This evidence-based approach may advocate for broader adoption and funding of similar programs globally, ultimately contributing to the reduction of NCDs on a larger scale.

One strength of this study is its large sample size and randomized controlled design, which enhances the reliability and generalizability of the findings. Additionally, the use of the LS7 score and biometric parameters provides a comprehensive evaluation of the participants’ health and lifestyles. However, there are potential limitations to consider. First, we recognize that LS7 score consists of self-reported data on lifestyles and measured body biometrics. The reliance on self-reported data may introduce reporting bias for our primary outcome. Secondly, the effectiveness of HW is more likely to be affected by participant’s adherence and usage to the HW. Similarly, the interaction effect of HC + HW will also be affected. Nevertheless, we will provide technical support to participants and track whether the participants have used the HW. Future studies could explore strategies to enhance adherence and assess the long-term sustainability of the interventions.

## Dissemination of the results

Findings from the study will be submitted to open-access, peer-reviewed journals and international conference. The full study protocol will be made publicly available. However, there are no plans to grant public access to the participant-level dataset. Authorship for future publications will follow according to the International Committee of Medical Journal Editors (ICMJE) authorship criteria. The team does not plan to hire any professional writers.

## Ancillary studies

There will not be any ancillary studies.

## Conclusion

This trial will provide valuable insights into the effectiveness of HC and HW for promoting lifestyle changes among middle-aged individuals at-risk of NCD development. The findings could inform public health policies and contribute to the development of effective, evidence-based interventions to reduce the burden of chronic diseases.

## Trial status

Recruitment began on 1 March 2024 and ended on 30 September 2024. By 29 October 2024, 2 participants had completed the final assessments. Current submitted protocol version number is version 4, revised on date 15 October 2024. The updated protocol includes changes to the primary and secondary outcomes, as well as refinements to the descriptions of the inclusion and exclusion criteria. These outcome changes were made to better align with the overall purpose of our study, which is to evaluate the effectiveness of HC and HW, individually or in combination, in facilitating overall improvement in lifestyle that could prevent NCDs, rather than solely focusing on BP or biochemical outcomes. There have been no changes made to the eligibility criteria of the study since ethics approval. 

## Supplementary Information

Additional file 1: SPIRIT checklist

Additional file 2: Informed consent form for participants (only available in Chinese)

## Data Availability

No datasets were generated or analyzed during the current study.

## Abbreviations

AFPA: American Fitness Professionals and Associates
AHA: American Heart Association
AOBP: Automated office blood pressure
AUC: Area under the curve
AUDIT-C: Alcohol Use Disorders Identification Test-C
BMI: Body mass index
BP: Blood pressure
BSMAS: Bergen Social Media Addiction Scale
CBT: Cognitive behavioral therapy
Ch-AUDIT: Chinese-translated Alcohol Use Disorders Identification Test
COPD: Chronic obstructive pulmonary disease
CVD: Cardiovascular disease
DASH: Dietary Approaches to Stop Hypertension
DBP: Diastolic blood pressure
DM: Diabetes mellitus
FBG: Fasting blood glucose
GAD-7: Generalized Anxiety Disorder-7 Scale
HbA1c: Glycated hemoglobin
HC: Health coaching
HDL: High-density lipoprotein
HR: Heart rate
HT: Hypertension
HW: Health watch
ICMJE: International Committee of Medical Journal Editors
IPAQ-SF: International Physical Activity Questionnaire-Short Form
ITT: Intention-to-treat
LDL: Low-density lipoprotein
LE8: Life's Essential 8
LMMs: Linear mixed models
LS7: Life's Simple 7
MAR: Missing at random
MD: Mediterranean diet
MI: Motivational interviewing
NCDs: Non-communicable diseases
NGOs: Non-governmental organizations
NHS DPP: National Health Service Diabetes Prevention Programme
PCE: Pooled Cohort Equations
PHQ-9: Patient Health Questionnaire-9
PI: Principal investigator
PSQI: Pittsburgh Sleep Quality Index
PSS-10: 10-Item Perceived Stress Scale
PWV: Pulse wave velocity
RCT: Randomized controlled trial
SAEs: Serious adverse events
SBP: Systolic blood pressure
SD: Standard deviation
SPIRIT: Standard Protocol Items: Recommendations for Interventional Trials
SpO2: Oxygen saturation
TC: Total cholesterol
TG: Triglycerides
VAS: Visual analog scale
WHO: World Health Organization
