# Supplementary material for: Health coaching with physical monitoring using health wearable (HCHW) to prevent non-communicable diseases (NCDs) in the middle-aged: a 4-arm randomized controlled trial protocol
Source: Trials. 2025 Oct 8;26:394. doi: 10.1186/s13063-025-09081-5 (PMC12505725; doi:10.1186/s13063-025-09081-5)
Supplement: Supplementary file 2 — Additional file 2: Informed consent form for participants (only available in Chinese) [file 13063_2025_9081_MOESM2_ESM.docx]

**研究題目**

使用智能手錶及健康管理以預防中年人士患上慢性疾病：一項4臂隨機對照試驗

**研究背景及重要性**

慢性疾病是造成不良健康狀況、殘疾以及早逝的主要原因。根據世界衛生組織報告，慢性疾病的主要類型是心血管疾病（如心臟病發作和中風）、癌症、慢性呼吸系統疾病（如慢性阻塞性肺疾病和哮喘）和糖尿病。大多數慢性疾病是可以從生活方式來預防（例如健康飲食習慣、多做運動、減少吸煙和喝酒等）。慢性疾病預防的重點對象為中年人士，他們因著不同社會原因，如工作模式，生活作息等因素影響到日常生活方式，從而增加患上慢性疾病的風險。但因個別人士缺乏支持和動力去促進和維持健康行爲，改變生活方式是一項挑戰。唯傳統的生活行爲介入方法是基於醫學模型，未能考慮個別人士所面對的問題、心理上的準備，以及社會和環境的支持。

香港中文大學賽馬會公共衛生及基層醫療學院獲得香港賽馬會慈善信託基金資助，聯同本地四間社會服務機構—聖雅各福群會、基督教家庭服務中心、基督教聯合那打素社康服務及香港基督教女青年會聯合主辦這項4臂隨機對照試驗。此試驗目的為評估健康管理及健康手錶的效用，以分析健康管理及健康手錶能否幫助中年人士改善生活習慣，以達致預防慢性疾病，及改善身體健康狀況。

**研究目的**

這項計劃旨在評估健康管理及健康手錶的效用。

**合資格參加者的條件**

這項計劃將招募1,000名參加者。他們必須是

1. 年齡介乎35至59歲之間人士；
2. 沒有被醫生診斷患有下列慢性疾病或需要定期服藥（包括：糖尿病、高血壓、心血管疾病、慢性阻塞性肺病、中度或重度抑鬱症、免疫系統疾病、以及癌症）；
3. 未有因患上上述慢性疾病而正在輪候公立醫院，或私立醫院／診所的跟進服務；
4. 沒有正在參與其他機構提供的健康管理服務；
5. 能夠理解廣東話
6. 在研究期間並沒有懷孕。

**研究計劃內容**

這項計劃目的為評估健康管理及健康手錶的效用，研究時期為六個月。所有參加者均會接受健康管理及獲派發健康手錶。

評估將會在 1）研究開始前，以及 2）在研究開始後六個月進行。初步符合參加資格的參加者首先會進行健康風險篩選，再按照篩選結果評定患上慢性疾病風險。1) 風險較低的參加者可以維持現有生活方式；2) 若參加者接受血液及血壓測試後，發現確診糖尿病，高血脂或高血壓，參加者將獲得健康報告，以便尋求專業醫療協助；3) 若被評估為有較高慢性疾病風險，便要接受血液測試及血壓測試，以詳細評定患上慢性疾病風險的等級，若發現糖尿病或高血壓指標未達確診水平，將需要填寫詳細的基線調查問卷，並隨機分組到以下其中一組別:

第一組: 完成基線評估後，接受為期六個月的健康管理教練指導和使用健康手錶。

第二組: 完成基線評估後，接受為期六個月的健康管理教練指導，並在完成六個月評估後獲派發健康手錶。

第三組: 完成基線評估後，獲派發健康手錶。

第四組: 完成基線評估後先等候六個月，並在完成六個月評估後接受為期六個月的健康管理教練指導並獲派發健康手錶。

以下為計劃的詳細內容：

1. 健康管理教練

參加者將會與一名健康管理教練配對。健康管理教練先評估參加者生活方式，之後將會為其度身設計目標及行動計劃。在健康管理教練和參加者的共識下，參加者將執行行動計劃，健康管理教練會因應參加者情況調整行動計劃內容。此外，健康管理教練將會定期與第一及第二組參加者聯絡（單獨／群組面見或透過電話，通訊軟件），並提供健康管理指導。

1. 運動健康手錶

參加者會獲派發一隻運動健康手錶，以收集健康數據（包括：運動記錄、心率、睡眠、及血壓）。

1. 「CORE」活動

參加者會被安排參加一個「CORE」活動。包括以下三個範疇：1）運動；2）心理健康；3）營養。活動由社會服務機構提供。

1. 賽馬會「We WATCH」優活健康計劃應用程式 (下稱「應用程式」)

參加者可以從「應用程式」上得知個人健康資訊。健康管理教練可以透過「應用程式」向參加者更新生活方式及習慣的建議。參加者也可以從「應用程式」獲得有實證的生活健康資訊、經「應用程式」分享成果、以及報名參加與我們合作的社會服務機構所舉行的「CORE」活動。

**參加者好處**

研究人員不會向閣下索取任何費用。所有有較高慢性疾病風險的參加者均會獲得健康報告。

參加者在完成六個月計劃，以及完成所有階段的評估後，將可保留運動手錶。倘若參加者未能完成六個月計劃，及未能完成所有評估，中文大學有權透過社會服務機構要求參加者交回運動手錶，**中文大學保留最終決定權**。

**自願參與 / 中途退出**

是次研究的參與屬自願性質， 閣下有權選擇是否參與。 即使簽署了<參加者同意書>， 閣下亦有權拒絕回答問卷中任何問題，或隨時退出是次研究，而絕對不會影響 閣下現享有的醫療服務及法律權益。

參加者請注意，為免影響研究結果的公平性，在接受介入方法的六個月內，不可以參加其他研究計劃，否則會被即時退出本項目。

**保密性**

在你參與這研究期間所取得的資料包括錄音，影片都會被嚴格保密。若你決定參與本研究並簽署此同意書(如本同意書為電子版本，可以剔選相關選項代替簽署) ，即表示你批准大學指定研究人員和香港中文大學 - 新界東醫院聯網臨床研究倫理席委員會和管理當局在法律監督下檢閱你有關本研究的部份醫療與研究記錄，以作教育培訓，研究和道德倫理審查之用途。

為保護你的個人資料，你的個人資料，包括你的姓名、證件號碼（例如香港身份證）及電子聯繫的資料（如電話號碼、電郵地址）將被保密並保存最多十年，而不會披露於任何第三方。你的姓名/身份不會顯示在任何報告、刊物或將來被公開的資料。

有需要的話，每位研究參與者都有權利獲得其個人的數據以及公開報告的研究結果。

根據香港法律（特別是「個人資料（私隱）條例」，第486章），閣下有權對您個人資料進行保密，如在本項研究中或與本項研究有關的個人資料的收集、保管、保留、管理、控制、使用（分析或比較）、在香港內外轉讓、不披露、消除和/或任何方式處理。如有任何問題，閣下可以諮詢人資料私隱專員或致電到其辦公室（電話號碼：2827 2827），以適當監管或監督 閣下個人資料保護，以便 閣下能完全認識和瞭解確保遵守法律保護隱私資料的意義。

同意參與該項研究，您明確作出以下授權:

• 為了監督該項研究，授權主要研究者及其研究團隊和倫理委員根據本項研究和本知情同意書規定的方式獲得、使用並保留您的個人資料，並且

• 為了檢查和核實研究資料的完整性、評估研究協定與相關要求的一致性，授權相關的政府機構（如香港衛生署）可獲得您個人資料。

本研究負責人為葉漢基教授 (Prof. Benjamin YIP電郵：benyip@cuhk.edu.hk) ，如有任何查詢，歡迎致電本項目經理鄭繼璇小姐（Ms. Karen CHENG電郵： karenkscheng@cuhk.edu.hk；電話：2609 5126）。

若閣下對作為研究參與者所享有的權利有任何疑問，請致電 3505 3935香港中文大學 - 新界東醫院聯網臨床研究倫理席委員會聯絡。

感謝您考慮參與這項研究和問卷調查。如有任何有關研究倫理的問題，您可以聯絡香港中文大學調查及行為研究操守委員會查詢，電郵：fssc02@cuhk.edu.hk。

=========================================================================

參加同意書

□ 本人已經閱讀此同意書的有關此研究計劃之詳情，清楚了解計劃之一切程序，亦明白本人的約定及承擔。

□ 本人已經閱讀是次研究計劃的目的、方法及資料運用；所有收集得來的資料將由研究員保管，絕對保密，並且只作研究之用。

□ 本人同意在參加研究項目期間提供本人的血液樣本，作為研究項目的用途。

□ 本人知悉此研究所得的資料可能被用作日後的研究及發表，但本人的私隱權利將得以保留，即本人的個人資料不會被公開。

□ 本人同意中文大學未來在基於本項目的研究，與本人聯絡。

□ 本人在此聲明，本人自願參加賽馬會「We WATCH 」優活健康計劃。

姓名:__________________________

簽名:__________________________

日期:__________________________
